# Prevalence of Hypertension and Its Associated Factors among Adolescents in Eastern Sudan: A Community-Based Study

**DOI:** 10.3390/children11080888

**Published:** 2024-07-24

**Authors:** Saeed M. Omar, Ahmed A. Hassan, Abdullah Al-Nafeesah, Ashwaq AlEed, Jaber Alfaifi, Ishag Adam

**Affiliations:** 1 Faculty of Medicine, Gadarif University, Gadarif 32211, Sudan; drsaeedomar@yahoo.com (S.M.O.); ahmed.ahmed@research.sunderland.ac.uk (A.A.H.); 2 Department of Pediatrics, College of Medicine, Qassim University, Buraydah 52571, Saudi Arabia; a.alnafeesah@qu.edu.sa; 3 Department of Child Health, College of Medicine, University of Bisha, Bisha 67714, Saudi Arabia; jalfaifi@ub.edu.sa; 4 Department of Obstetrics and Gynecology, College of Medicine, Qassim University, Buraydah 52571, Saudi Arabia; ia.ahmed@qu.edu.sa

**Keywords:** hypertension, age, blood pressure, adolescents, body mass index, obesity

## Abstract

Background: Elevated blood pressure, or hypertension, is one of the main health problems among adolescents globally. However, there are limited data on hypertension among adolescents in Sudan. This survey aimed to investigate the prevalence of elevated blood pressure/hypertension and associated factors among adolescents in Gadarif City, Sudan. Methods: A community-based cross-sectional survey was conducted during a three-month period (August to October 2023) in Gadarif City, Eastern Sudan. A face-to-face interview questionnaire was used to collect sociodemographic information. Adolescents’ anthropometric (weight and height) measurements were taken, and blood pressure was measured. Multivariate binary and linear regression analyses were performed to analyze the data. Results: A total of 384 adolescents (178 [46.4%] boys and 206 [53.6%] girls) were included in the study. The median (interquartile range, IQR) of the age was 14.0 (12.1–16.1) years, and that of the body mass index (BMI) was 16.9 (15.2–20.0) kg/m^2^. Thirty-four (8.9%) adolescents had hypertension/elevated blood pressure (≥95th percentile). After adjusting for confounders, multivariable binary regression analysis showed that age (adjusted odds ratio [AOR], 1.20; 95% confidence interval [CI], 1.03–1.42) and BMI (AOR, 1.12; 95% CI, 1.04–1.20) were associated with hypertension. Conclusion: Approximately one in ten adolescents in Eastern Sudan was hypertensive. Adolescents with higher age and BMI were at higher risk for hypertension. Maintaining a healthy BMI during adolescence is recommended to promote adolescents’ health.

## 1. Introduction

Hypertension/elevated blood pressure is one of the main worldwide health problems in children and adolescents, and Sub-Saharan Africa (SSA) is no exception [1,2,3,4]. There are 26.5 million adolescents in SSA who have elevated BP, and these numbers are expected to increase [5]. Researchers attribute this trend to the increasing prevalence of overweight and obesity among children and adolescents [1,2]. Adolescent obesity is associated with comorbidities, including metabolic syndrome and cardiovascular disease [6]. Adolescence is a crucial period in human development and plays a significant role in future health outcomes [7]. The “World Health Organization (WHO)” defines adolescence as “the phase of life between childhood and adulthood, extending from ages 10 to 19 years” [7].

Recent reports have shown various rates of hypertension/elevated blood pressure among children and adolescents in SSA [1,2,8,9,10,11,12,13,14,15,16,17,18]. For example, in their systematic review, Nsanya et al. reported a widely varied (0.18% to 34.0%) prevalence of hypertension/elevated blood pressure among adolescents in SSA [18].

Several risk factors, such as being male, older age groups, low paternal education level, having a positive family history of hypertension, smoking [1,2,14,15], overweight, and obesity [1,2,14,15,19], have been observed to be associated with hypertension among children and adolescents.

While hypertension among adults and its complications receive more attention, adolescents’ hypertension tends to be overlooked. To take a more proactive approach, hypertension among children and adolescents needs to be addressed by early assessment of its prevalence and associated factors, particularly within the community, and by implementing appropriate intervention measures accordingly. This approach could help to detect, manage, and prevent the development of hypertension and its associated complications (such as cardiovascular disorders) in later adulthood [3,20]. Moreover, screening blood pressure at an early age, that is, in children, adolescents, and young adults, provides opportunities to address, prevent, and detect complications such as cardiovascular disorders and manage secondary causes of hypertension, including endocrine and renal diseases [21]. Such preventive measures should be encouraged, especially in contexts such as Sudan, where resources for management are limited and poor. Additionally, adolescents represent a significant proportion of Sudan’s population, with the WHO estimating that more than 20% of Sudanese are adolescents [22].

Few studies have addressed the epidemiology (prevalence and associated factors) of hypertension among adolescents in Sudan [19,23], and no such studies have been conducted in Eastern Sudan. Moreover, we have previously shown that 40.8% of the adults in Eastern Sudan had hypertension [24,25]. This study investigated the prevalence of elevated blood pressure/hypertension among adolescents in Gadarif City, Sudan, and assessed the possible associated factors.

## 2. Materials and Methods

### 2.1. Study Design and Setting

A community-based cross-sectional survey was conducted among 384 adolescents in Gadarif City, Eastern Sudan, from August to October 2023. Gadarif State is located east of Sudan, neighboring Ethiopia, with Gadarif City serving as its capital. According to the 2008 census, the total population of Gadarif State was 1,400,000 [26]. There are 11 localities in Gadarif State, and the Gadarif locality (where Gadarif City is located) is the most populated. Gadarif State has expansive agricultural land and hosts Sudan’s most significant projects for rain-fed agriculture. The region encompasses diverse ethnic groups, reflecting a multiethnic society [27]. More details about the study area were reported in our previous study [28]. In the present study, we strictly adhered to the “Strengthening the Reporting of Observational Studies in Epidemiology (STROBE) guidelines” [29].

### 2.2. Sampling Technique

The Gadarif locality was chosen from the 11 localities due to its diverse population, which could effectively represent the entirety of Gadarif State. Gadarif City is divided into four zones (Mouraba), each comprising 13 blocks (Hay). The total population in each block was obtained from local authorities. Estimating that adolescents make up approximately 20% of the total population, in accordance with the WHO’s estimation [22], a sample size of 384 adolescents, including males and females, was targeted. The number of adolescents selected from each block depended on the proportionate representation of the total estimated adolescent population in that specific block (i.e., probability proportional to size). The list of households was selected from each block using a simple random technique (lottery method). If a chosen household had no adolescents, refused to participate in the study, or met any exclusion criteria, the next household was approached for replacement.

### 2.3. Inclusion and Exclusion Criteria

The inclusion criteria included healthy adolescents aged 10–19 years. Participants outside this age range (<10 years or >19 years), individuals who declined consent for participation, those with known hypertension, individuals who were sick, pregnant adolescents, or those who were lactating were excluded from the study.

### 2.4. Sample Size Calculation

OpenEpi Menu software Version 3.01 was used to compute the required sample size, which is 384 adolescents [30]. Given the absence of previous studies in the study region, the maximum event occurrence (hypertension) was assumed to be 50.0% to determine the optimal sample size using the equation n = Z2pq/d2, where q = (1 − p), Z1 − α = confidence interval (CI) of 95% = 1.96, and d = margin of error of 5% = 0.05.

### 2.5. Study Variables and Measures

The questionnaire was designed based on previously conducted studies in different regions of Sudan [1,2,12,14,15,19,31]. It included data on sociodemographic characteristics such as age (in years), sex (male or female) of the adolescents, parents’ educational levels, which was divided as <secondary or ≥secondary, mother’s occupational status (housewife or employed), father’s occupational status (employed or manual worker), cigarette smoking habit, and family history of hypertension. Additionally, anthropometric measurements, such as weight and height (later expressed as body mass index [BMI]), were included, along with hypertension status. The authors trained five medical officers to collect the data.

Upon obtaining informed consent from participants and their guardians, the medical officers approached the selected adolescents, briefed them about the study’s objectives, and provided all necessary information, including adolescents’ voluntary participation and their right to withdraw their consent. All necessary preventive measures were taken to ensure the participants’ privacy, confidentiality, and safety, including excluding adolescents’ personal identifiers during data collection. Weight, height, and blood pressure were measured using the standard procedures outlined below. Sociodemographic information and BMI were considered secondary outcomes, while hypertension was the primary focus of the study.

### 2.6. Weight and Height Measurements

The adolescent’s weight was measured in kilograms (kg) using standard procedures, which included using well-calibrated scales adjusted to zero before each measurement. Weight was recorded to the nearest 100 g. During measurement, adolescents were asked to remove their shoes and excess clothing and stand still with minimal movement, keeping their hands by their sides. Height was measured to the nearest 0.1 cm and later computed to meters (m), with the adolescents standing straight with their backs against the wall and feet together. BMI was computed using the following formula: weight in kg divided by height in m^2^ [32]. To accurately estimate the influence of each one-unit increase in BMI on blood pressure among adolescents, this study analyzed BMI as a continuous variable, similar to recently published studies [14,33].

### 2.7. Blood Pressure Measurement

After a minimum resting period of 10 min, two blood pressure readings were taken using a standardized digital blood pressure measuring device “(Omron Digital HEM-907, Tokyo, Japan)”, with the arm maintained at heart level. Then, the average of the two blood pressure readings was computed. If the difference between the two readings exceeded 5 mmHg, additional measurements were taken until the readings stabilized. Systolic and diastolic blood pressures were computed according to age and sex, following guidelines for diagnosing, evaluating, and treating hypertension in children and adolescents [34]. Hypertension in this study was defined as average systolic and diastolic pressures ≥ 95th percentile for age, sex, and height. The definition outlined by the American Academy of Pediatrics in 2017 [34], widely reported and utilized for diagnosing hypertension in children and adolescents across Africa [1,2], was employed in this study.

### 2.8. Statistical Analysis

The data were analyzed using IBM Statistical Product and Service Solutions (SPSS) for Windows (version 22.0; SPSS Inc., New York, NY, USA). The Shapiro–Wilk test was used to assess the normality of the continuous variables, namely age, BMI, pulse blood pressure, and mean blood pressure. They were found to have a non-normal distribution. These variables (age, BMI, pulse pressure, and mean blood pressure) were expressed as median with interquartile range (IQR). A univariate (binary and linear) analysis was conducted with hypertension, pulse pressure, and mean blood pressure as the dependent variables, and the independent variables were age, sex, BMI, parental education level and occupation, smoking habit, and family history of hypertension. Subsequently, variables with a *p*-value < 0.20 in univariate analysis were shifted to build a multivariable (binary and linear) regression model to adjust for covariates. Adjusted odds ratios (AORs) with their corresponding 95% CIs were calculated, with a *p*-value < 0.05 considered statistically significant. Pearson correlation was performed between continuous variables, and its coefficient (r) value (rather than the *p*-value) was considered strong if it was greater than 0.5 and moderate if it was between 0.3 and 0.5.

## 3. Results

Out of the total sample of 384 adolescents, 178 (46.4%) were boys and 206 (53.6%) were girls. The median (IQR) age and BMI were 14.0 (12.1–16.1) years and 16.9 (15.2–20.0) kg/m^2^, respectively. Among the adolescents, 267 (69.5%) had mothers with a secondary education level or above, and 288 (75.0%) had fathers with a secondary education level or above. Approximately 1 in 5 adolescents (71, 18.5%) had mothers who were employed. A small proportion of adolescents (6.6%) were cigarette smokers, and 116 (30.2%) had a family history of hypertension (Table 1).

The median (IQR) of systolic blood pressure was 116 mmHg (110–122 mmHg), and that of diastolic blood pressure was 79 mmHg (70–80 mmHg). Thirty-four adolescents (8.9%) had hypertension (systolic and diastolic pressures ≥ 95th percentile).

In the univariate analysis, increasing age (unadjusted OR, 1.23; 95% CI, 1.11–1.50) and increasing BMI (unadjusted OR, 1.16; 95% CI, 1.08–1.24) were associated with hypertension, while sex, parental education level and occupation, cigarette smoking habit, and family history of hypertension showed no significant association (Table 1).

In the multivariable logistic regression analysis, increasing age (AOR, 1.20; 95% CI, 1.03–1.42) and BMI (AOR, 1.12; 95% CI, 1.04–1.20) remained associated with hypertension (Table 2).

In univariate and multivariate linear regression, age and BMI were associated with pulse and mean arterial pressure (Table 3 and Table 4).

As per the coefficient (r) value, there was a strong positive correlation between systolic, diastolic, pulse, and mean blood pressure. There was a strong positive correlation between diastolic and mean blood pressure. There was a moderate positive correlation between age and BMI; between BMI, systolic, and mean blood pressure; and between pulse and mean blood pressure (Table 5).

## 4. Discussion

The primary finding of this study was that 8.9% of adolescents in Eastern Sudan suffered from hypertension, with increasing age and BMI showing significant associations with hypertension. This prevalence of 8.9% aligns with findings from similar studies conducted in Sudan and other countries [15,19]. For instance, our previous school-based study in Northern Sudan revealed that approximately 10% of adolescents reported hypertension [19]. In Tanzania, a survey among children aged 6 to 17 years found a hypertension prevalence of 10.8% [15]. Additionally, a recent systematic review and meta-analysis of 36 studies encompassing 37,926 adolescents aged 10 to 19 years across Sub-Saharan Africa revealed that one in ten adolescents exhibited elevated blood pressure [4]. However, the current prevalence of 8.9% is slightly higher than the rates reported in previous studies in various African countries [9,14,16]. For instance, in Ghana, 6.0% of adolescents were found to have pre-hypertension, and 2.5% were diagnosed with hypertension [18]. Similarly, in Uganda, 3.1% of adolescents had hypertension [9], and in South Africa, 5.2% of adolescents suffered from hypertension [14].

Conversely, the prevalence of hypertension observed in this study (8.9%) is lower than rates reported in countries such as Tunisia (15.4%) [12] and Nigeria, where the prevalence ranged from 12.7% to 19.0% [11]. Almahmoud et al., in their systematic review and meta-analysis, found a high prevalence of hypertension (12.6%) and pre-hypertension (13.9%) among adolescents across Arab countries [20], with prevalence rates about four times higher in high-income countries like Saudi Arabia than in middle-income countries like Tunisia and Egypt. Furthermore, prevalence was found to be higher among adolescents with an increased BMI [20].

As already mentioned, a recent systematic review encompassing 92 studies revealed a significant disparity in the prevalence of hypertension among adolescents in Sub-Saharan Africa, ranging from 0.18% to 34.0% [18]. Similarly, Almahmoud et al., in their systematic review and meta-analysis, reported wide variations in hypertension prevalence across Arab countries, with rates ranging from 4% to 26% [20]. These variations underscore the importance of investigating the epidemiology of hypertension within specific countries or regions to develop tailored strategies for combating this health issue.

Several factors contribute to the high prevalence of hypertension among adolescents. Sudanese adolescents from urban areas, such as Gadarif City, exhibited increased obesity rates, lack of physical activity, and sedentary behaviors, such as prolonged television viewing and internet usage [35]. Moreover, urban-dwelling adolescents tend to have a high prevalence of hypertension compared to their counterparts in semi-urban or rural areas [12].

This elevated prevalence of hypertension among adolescents (8.9%) may explain the high prevalence of hypertension and its complications among adults in Eastern Sudan, as indicated in our previous studies [24,25], that is, neglecting early preventive measures against hypertension during childhood and adolescence may contribute to hypertension development in adulthood. Robinson et al., in their review, emphasized the importance of early screening and management of childhood hypertension to improve future kidney and cardiovascular health outcomes [36]. Similarly, in South Africa, Raphadu et al. recommended blood pressure and body weight screening for adolescents, especially those with pre-hypertension, who are at heightened risk of developing hypertension [31].

In this study, we found that increased age was associated with hypertension among adolescents; specifically, an increase of one year in age enhanced the risk of hypertension by 1.12 times. This translates to a 20% increase in the likelihood of hypertension for each additional year of age among adolescents. Consistent with our findings, several studies conducted in different countries have also identified a significant association between advancing age and hypertension [12,14,37]. For instance, a cross-sectional study conducted in Cameroon among children and adolescents aged 3 to 19 years revealed that adolescents over the age of 14 years were three times more likely to have hypertension [12]. Similarly, in South Africa, a cross-sectional study including 876 children aged 9 to 14 years showed a significant correlation between increasing age and higher BMI with hypertension [14]. In India, a community-based cross-sectional study of 550 adolescents found that those aged 15–19 years and those classified as overweight or obese were at two-and-a-half and four times higher risk of developing hypertension, respectively [37].

However, it is worth noting that some studies, including those conducted in Sudan, have reported no significant association between age and hypertension among adolescents [10,19,38]. Interestingly, a study of 16,182 adolescents aged 10 to 19 years in India by Vasudevan et al. contradicted our findings, revealing a high prevalence of hypertension among young adolescents aged 10 to 12 years (35.1%) and adolescents aged 13 years or above (25.1%) [39]. Furthermore, the study highlighted that adolescents classified as overweight or obese were at a higher risk of developing high blood pressure [39].

This study revealed that an increased BMI was significantly associated with hypertension among adolescents. Specifically, adolescents with each additional BMI kg/m^2^ were found to be 1.12 times more likely to have hypertension; that is, a one-unit increase in BMI contributed to a 12% higher risk of hypertension among adolescents. Similar findings have been reported in studies from various countries, including Sudan, highlighting a significant positive association between a higher BMI and hypertension among adolescents [10,14,33,39,40,41]. Our previous school-based cross-sectional study conducted in Northern Sudan involving 384 adolescents revealed a similar AOR, indicating that an increasing BMI (AOR, 1.12) was associated with hypertension [19]. Likewise, a study in South Africa involving 1665 children and adolescents aged 5 to 15 years found that a one-unit increase in BMI kg/m^2^ was associated with a 1.20 times and 1.23 times higher risk of systolic and diastolic hypertension, respectively [33]. Another study in South Africa, which included 876 children aged 9–14 years, revealed a significant association between increased BMI and elevated blood pressure in children and adolescents (AOR, 1.06, 95% CI, 1.02–1.11) [14]. In Central Sudan, Elfaki et al. revealed that early adolescents with obesity were at a higher risk of developing metabolic syndrome, including hypertension, compared to those with an average weight or overweight [23]. Similarly, in a recent large-scale cross-sectional study involving 1385 Tunisian adolescents, Soua et al. found that those who were overweight and obese had two times and four times higher risk of developing hypertension, respectively [10]. In a recent systematic review and meta-analysis, Noubiap et al. concluded that overweight and obesity were the main risk factors for the high prevalence of elevated blood pressure among children and adolescents in Africa; specifically, children and adolescents with obesity were found to have six times higher risk of developing elevated blood pressure in Africa [2].

This study found no significant association between adolescents’ hypertension and factors such as sex, parental education level and occupation, and family history of hypertension. The absence of such associations can be explained by several factors. For example, the lack of association with parental education could stem from the generally low quality of education, especially among mothers, and its limited impact on workforce participation. In this study, only about 20% of mothers were employed, which aligns with previous findings indicating a low maternal employment rate (10%) in Eastern Sudan [42].

Similarly, the lack of association with a family history of hypertension corroborates the findings from our previous study in Northern Sudan [19]. In contrast, other studies have reported associations between family history and adolescent hypertension [1,2,16,17]. However, in our context (Northern and Eastern Sudan), the absence of such associations suggests that environmental and lifestyle factors may play more significant roles in developing hypertension than genetic factors.

The lack of association between adolescent sex and hypertension in this study is consistent with previous studies conducted in various African countries, including Sudan [2,14,15,19]. In contrast, studies from Nigeria and Cameroon have identified being female as a risk factor for adolescent hypertension [12,19]. Additionally, Azupogo et al. reported that being male was a risk factor for adolescent hypertension in Ghana [13].

The present findings warrant a cautious comparison with those of other studies for various reasons. First, the present study adhered to the WHO age cut-off (10–19 years). In contrast, other studies may have employed different age ranges, such as early adolescence, late adolescence, or a combination of adolescents and children. Second, variations in sociodemographic characteristics, such as urban residency and smoking habits, across different settings should be considered. For instance, research in Nigeria by Ayogu and Nwodo showed a positive association between smoking habits and the development of hypertension among adolescents [11].

These findings have implications for improving adolescent health, as one key identified factor—BMI—can be modified through lifestyle changes. Launching school-based and community-based initiatives to promote physical activity and a healthy diet is recommended to maintain a healthy BMI during adolescence and adulthood. Such initiatives are crucial for maintaining adolescents’ health in their present and later lives. The study findings and these recommendations should be shared with policymakers for incorporation into existing policies targeting adolescent health.

## 5. Strengths and Limitations of the Study

To our knowledge, this study is the first to explore the epidemiology of hypertension among adolescents in Eastern Sudan. This adds significant value to the limited existing research on elevated blood pressure/hypertension among adolescents in Sudan [19,23]. The recommendations outlined in this study can be utilized by stakeholders to improve children’s and adolescents’ health in Sudan, particularly given the ongoing war and its adverse impacts on the health of vulnerable populations, especially children and adolescents [43]. However, it is important to acknowledge certain limitations of this study. First, this was a cross-sectional study; a longitudinal study will provide more insights into the association between hypertension and various nutritional status variables among adolescents. Second, this study was conducted among adolescents in one region of Sudan (Eastern Sudan), which may limit the generalization of its results to adolescents in other regions of Sudan. Additionally, data on ethnicity, race, socioeconomic status, dietary patterns, salt intake, and lifestyle practices that can influence blood pressure were not collected in this study [8]. Moreover, 24 h ambulatory blood pressure monitoring was not performed.

## 6. Conclusions

Approximately one in ten adolescents in Eastern Sudan was hypertensive, with increasing age and BMI showing significant associations with hypertension. To promote the well-being of adolescents in their present and later lives, initiatives focused on maintaining a healthy BMI during adolescence are recommended. The involvement of all related stakeholders is crucial for the sustainability of such initiatives.

## Figures and Tables

**Table 1:** Characteristics and univariate binary analysis of the factors associated with hypertension in adolescents in Eastern Sudan (N = 384), 2023.

Variable		Total (N = 384)	Adolescents with Hypertension (34/384 = 8.9%)	Adolescents without Hypertension (350/384 = 91.1%)	Unadjusted Odds Ratio (95% Confidence Interval)	*p*-Value
		Median (Interquartile range)				
Age, years		14.0 (12.1–16.1)	16.1 (14.2–17.5)	13.8 (12.0–15.9)	1.23 (1.11–1.50)	0.001
Body mass index, kg/m^2^		16.9 (15.2–20.0)	21.1 (17.6–25.0)	16.7 (15.1–19.5)	1.16 (1.08–1.24)	<0.001
		Frequency (proportion)				
Gender	Male	178 (46.4)	16 (47.1)	162 (46.3)	Reference	
	Female	206 (53.6)	18 (52.9)	188 (53.7)	0.97 (0.48–1.96)	0.930
Mother education level	≥secondary	267 (69.5)	23 (67.6)	244 (69.7)	Reference	
	<secondary	117 (30.5)	11 (32.4)	106 (30.3)	1.10 (0.52–2.34)	0.803
Father education level	≥secondary	288 (75.0)	29 (85.3)	259 (74.0)	Reference	
	<secondary	96 (25.0)	5 (14.7)	91 (26.0)	0.49 (0.18–1.31)	0.154
Mother occupation	Employed	71 (18.5)	6 (17.6)	65 (18.6)	Reference	
	Housewife	313 (81.5)	28 (82.4)	285 (81.4)	1.06 (0.42–2.68)	0.895
Father occupation	Employed	148 (38.5)	20 (58.8)	216 (61.7)	Reference	
	Manual worker	236 (61.5)	14 (41.2)	134 (38.3)	0.89 (0.43–1.81)	0.741
Cigarette smoking	No	378 (98.4)	33 (97.1)	345 (98.6)	Reference	
	Yes	6 (1.6)	1 (2.9)	5 (1.4)	2.09 (0.24–18.43)	0.507
Family history of hypertension	No	268 (69.8)	22 (64.7)	246 (70.3)	Reference	
	Yes	116 (30.2)	12 (35.3)	104 (29.7)	1.29 (0.62–2.70)	0.500

**Table 2:** Multivariable binary regression analysis of factors (adjusted) associated with hypertension among adolescents in Eastern Sudan (N = 384), 2023.

Variable		Adjusted Odds Ratio (95% Confidence Interval)	*p*-Value
Age, years		1.20 (1.03–1.42)	0.022
Body mass index, kg/m^2^		1.12 (1.04–1.20)	0.002
Father education	≥secondary	Reference	0.288
	<secondary	0.58 (0.21–1.58)	

**Table 3:** Univariate linear analysis of the factors associated with pulse pressure and mean blood pressure in adolescents in Eastern Sudan (N = 384), 2023.

Variable		Pulse Pressure			Mean Arterial Pressure		
		Coefficient	95.0% Confidence Interval	*p*-Value	Coefficient	95.0% Confidence Interval	*p*-Value
Age, years		0.63	0.27–0.98	0.001	0.68	0.35–1.01	0.001
Body mass index, kg/m^2^		0.44	0.25–0.64	0.001	0.61	0.43–0.79	0.001
Gender	Male	Reference			Reference		
	Female	−0.048	−2.2–1.14	0.597	1.54	−0.11–3.21	0.068
Mother education level	≥secondary	Reference			Reference		
	<secondary	1.82	−0.11–3.76	0.066	0.94	−0.86–2.75	0.304
Father education level	≥secondary	Reference			Reference		
	<secondary	−0.23	−2.3–1.84	0.827	2.13	0.20–4.03	0.030
Mother occupation	Employed	Reference			Reference		
	Housewife	2.31	0.01–4.61	0.051	0.15	−1.99–2.30	0.889
Father occupation	Employed	Reference			Reference		
	Manual worker	1.53	−0.30–3.36	0.102	0.99	−0.71–2.70	0.253
Cigarette smoking	No	Reference			Reference		
	Yes	−1.11	−8.30–6.11	0.762	0.20	−6.52–6.93	0.952
Family history of hypertension	No	Reference			Reference		
	Yes	0.64	−1.30–2.60	0.514	2.26	0.46–4.06	0.014

**Table 4:** Multivariate linear regression of the factors associated with pulse pressure and mean arterial pressure in adolescents in Eastern Sudan (N = 384), 2023.

Variable		Mean Blood Pressure			Pulse Pressure		
		Coefficient	95.0% Confidence Interval	*p*-Value	Coefficient	95.0% Confidence Interval	*p*-Value
Age, years		0.33	0.001–0.67	0.049	0.40	0.02–0.77	0.036
Body mass index, kg/m^2^		0.51	0.32–0.70	0.001	0.37	0.16–0.58	0.001
Father education level	≥secondary	Reference					
	<secondary	1.53	−0.28–3.34	0.098			
Family history of hypertension	No	Reference					
	Yes	1.20	−0.52–2.94	0.171			

**Table 5:** Spearman correlation between age, body mass index, systolic, diastolic, pulse, and mean arterial pressure among adolescents in Eastern Sudan N = 384), 2023.

Variable	Body Mass Index, kg/m^2^		Systolic Blood Pressure, mm/Hg		Diastolic Blood Pressure, mm/Hg		Pulse Pressure		Mean Arterial Pressure, mm/Hg	
	*R*	*p*-Value	*R*	*p*-Value	*R*	*p*-Value	*R*	*p*-Value		
Age, years	0.421	<0.001	0.228	<0.001	0.154	0.003	0.150	0.003	0.210	<0.001
Body mass index, kg/m^2^			0.341	<0.001	0.200	<0.001	0.233	<0.001	0.384	0.291
Systolic blood pressure, mm/Hg					0.619	<0.001	0.703	<0.001	0.888	<0.001
Diastolic blood pressure, mm/Hg							−0.027	0.596	0.890	<0.001
Pulse pressure									0.354	<0.001

## Data Availability

The data supporting the current study’s findings are available from the corresponding author upon reasonable request.
